# Renal Angiomyolipoma with Tumor Thrombus in the Inferior Vena Cava and Right Atrium Accompanied by Renal Cell Carcinoma: A Case Report

**DOI:** 10.3390/medicina60081293

**Published:** 2024-08-10

**Authors:** Fan Shu, Yichang Hao, Ye Yan, Min Lu, Lulin Ma, Shaohui Deng, Liyuan Ge, Shudong Zhang

**Affiliations:** 1 Department of Urology, Peking University Third Hospital, Beijing 100191, China; 2 Department of Pathology, Peking University Third Hospital, Beijing 100191, China; 3 Department of Pathology, School of Basic Medical Sciences, Peking University Health Science Center, Beijing 100083, China

**Keywords:** renal angiomyolipoma, renal cell carcinoma, tumor thrombus, inferior vena cava, case report

## Abstract

*Background:* Renal angiomyolipoma (AML) without local invasion is generally considered benign. However, it may extend to the renal sinus, even the renal vein, or the inferior vena cava (IVC). In patients with non-tuberous sclerosis complex, coexistence of renal cell carcinoma (RCC) and renal AML is uncommon. *Case presentation:* A 72-year-old woman was incidentally found to have a solitary right renal mass with an IVC thrombus extending into the right atrium during a routine health checkup. Robot-assisted laparoscopic radical nephrectomy and thrombectomy were successfully performed through adequate preoperative examination and preparation. Two tumor lesions were found and pathologically confirmed as renal AML and RCC, and the tumor thrombus was derived from the renal AML. During the one-year follow-up period, no signs of recurrence or metastatic disease were observed. *Conclusions:* Renal AML with a tumor thrombus in the IVC and right atrium accompanied by RCC may occur, although rarely. In clinical practice, if preoperative manifestations differ from those of common diseases, rare diseases must be considered to avoid missed diagnoses. In addition, adequate examination and multidisciplinary discussions before making a diagnosis are necessary. For a level 4 tumor thrombus with no infringement of the venous wall, adoption of robot-assisted minimally invasive surgery, without extracorporeal circulation technology, is feasible.

## 1. Introduction

Renal angiomyolipoma (AML), also known as hamartoma, is a benign mesenchymal tumor composed of adipose tissue, blood vessels, and smooth muscle [1]. AML is derived from perivascular epithelioid cells, predominantly in young women [2], and may occur as an isolated phenomenon or as part of a syndrome associated with tuberous sclerosis (TS) [3]. TS is a rare multisystem, autosomal dominant disorder characterized by hamartoma of the brain, heart, kidney, skin, and lung [4], and the typical triad of TS is mental retardation, epilepsy, and the Pringle type of sebaceous adenoma (angiofibroma) [5]. Approximately 20% of AML cases are associated with TS and are usually bilateral and multifocal [6]. Most patients with renal AML are asymptomatic and tumors are often found incidentally during physical examinations [4]. With the constant improvement in imaging examinations, the detection rate of renal AML has increased. The classic renal AML is rich in fat and presents unique characteristics in computed tomography (CT) [7]. At present, the clinical diagnosis of renal AML mainly depends on pathological examination and signs of the expression of HMB45 [8]. Active surveillance is the accepted management for small asymptomatic masses. Other options for treatment include nephrectomy, selective arterial embolization, ablative therapies, and mTOR inhibitors [9]. Previous studies have reported no cases of widespread metastasis, but regional lymph node involvement has been documented [1].

Kutcher et al. reported the first AML presenting with a venous tumor thrombus in 1982 [10]. Since then, cases of AML with a renal vein or inferior vena cava (IVC) tumor thrombus have been reported [8,11,12]. In addition, simultaneous unilateral existence of AML and a renal cell carcinoma (RCC) is uncommon [12]. Here, we report a case of a tumor thrombus derived from AML involving the renal vein and IVC with concurrent RCC. Radical nephrectomy and thrombectomy remain the standard of care for renal tumors with tumor thrombus [13]. For patients with a right atrial tumor thrombus, the cardiopulmonary bypass is preferred [14]. In our case, the tumor thrombus had extended into the right atrium, and we performed a robot-assisted minimally invasive procedure to completely remove the tumor thrombus without the use of cardiopulmonary bypass, which minimized trauma to the patient and accelerated postoperative recovery.

## 2. Case Report

### 2.1. Medical History

A 72-year-old woman was hospitalized on 10 June 2022 because of a right renal mass found during an annual routine examination. There was no gross hematuria, lower back pain, abdominal pain, or symptoms of frequent urination, urgency, or dysuria. She had hypertension for more than 20 years and coronary heart disease for 10 years, and had been taking aspirin and atorvastatin for 10 years.

### 2.2. Physical Examination and Laboratory Testing

Physical examination revealed no percussion pain in the bilateral renal areas, no palpable mass, and no tenderness in the bilateral ureteral areas. There were no apparent abnormalities in laboratory testing, including routine blood, blood biochemistry, routine urine, and coagulation function tests.

### 2.3. Imaging Examination

**Figure 1:**
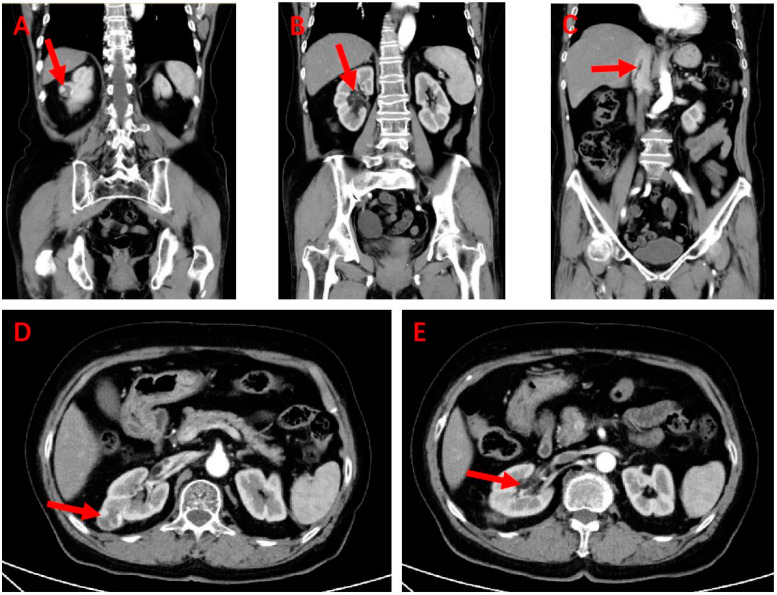
Abdominal contrast-enhanced computed tomography. (**A**) Rounded and high-density region (red arrow, renal cell carcinoma) measuring approximately 2.5 cm × 2.2 cm × 2.1 cm near the capsule area of the kidney (coronal plane). (**B**) Hilar angiomyolipoma lesion (red arrow) with a density close to that of adipose tissue, which was not identified preoperatively (coronal plane). (**C**) Low-density, linearly elongated tumor thrombus (red arrow) in the inferior vena cava (coronal plane). (**D**) Renal cell carcinoma lesion (red arrow) near the capsule of the kidney (horizontal plane). (**E**) Angiomyolipoma lesion (red arrow) in the renal hilum (horizontal plane).

**Figure 2:**
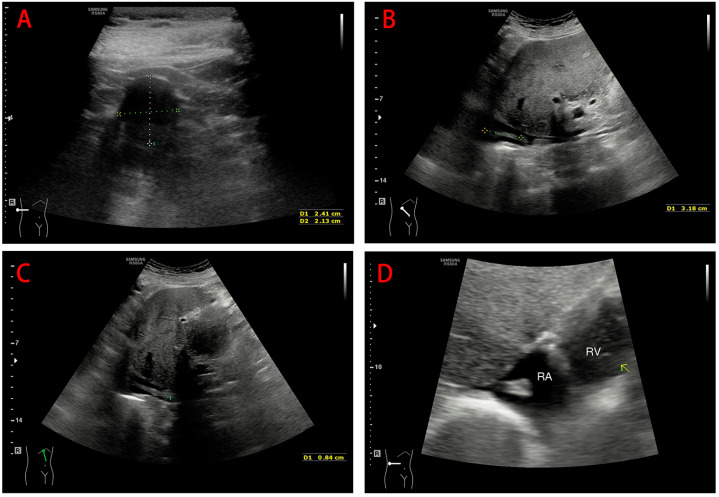
Color ultrasound Doppler of the inferior vena cava. (**A**) Hypoechoic nodule with a cross-sectional area of 2.41 cm × 2.13 cm in the right kidney (The colored dashed lines represented the two diametral lines of a cross section in different directions). (**B**) Linearly slender tumor thrombus along the direction of the extension of the inferior vena cava (The colored dashed line marked a distance traveled by the tumor thrombus). (**C**) The maximum diameter (colored dashed line) of the tumor thrombus in the inferior vena cava is approximately 0.8 cm. (**D**) Tumor thrombus protruding into the right atrium, with a large, round, and blunt tail (yellow arrow); RA: right atrium, RV: right ventricle.

Abdominal contrast-enhanced CT (Figure 1) showed an iso-intense ellipsoidal nodule with an unclear boundary in the unenhanced phase, measuring approximately 2.5 cm × 2.2 cm × 2.1 cm. During the arterial phase, the mass showed heterogeneous hyperenhancement relative to the adjacent renal parenchyma (Figure 1A,D). From the renal vein to the IVC (about the level of the second porta hepatis), a strip-shaped homogeneous hypodense shadow with a CT value of −70 HU was noted, which did not show significant enhancement in the arterial phase (Figure 1C). An additional video file shows this in more detail (Video S1). Ultrasound color Doppler of the IVC revealed a thrombus within, with a diameter of 0.5 cm and a length of approximately 9 cm, reaching up to the right atrium (Figure 2). The tip of the thrombus floated with a dense echo, which was round and blunt. Preoperative transesophageal ultrasonography showed that the tumor thrombus did not adhere to the wall of the IVC. 

### 2.4. Therapeutic Regimen

A multidisciplinary discussion was conducted involving several departments, including urology, anesthesiology, interventional vascular surgery, cardiac surgery, cardiovascular medicine, radiology, and ultrasound. We performed robot-assisted transabdominal laparoscopic radical nephrectomy of the right kidney and removal of the IVC tumor thrombus. Intraoperative transesophageal ultrasound was performed to dynamically visualize the actual tip position of the tumor thrombus and monitor embolization events. After sufficient dissociation of the kidney, the IVC wall was incised. The tumor thrombus did not invade the vein wall, and there was no adhesion. The tumor thrombus was quickly removed from the vessel (Video S2).

### 2.5. Postoperative Pathology

**Figure 3:**
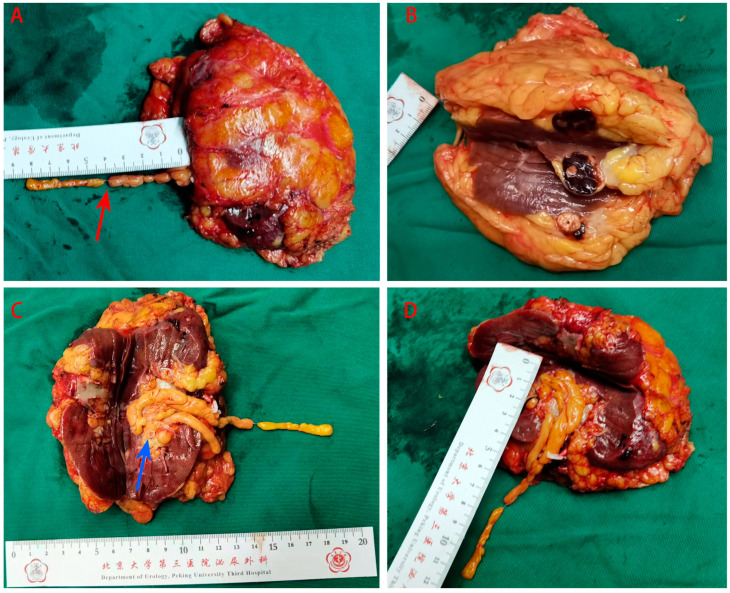
Kidney and tumor thrombus specimens. (**A**) Intact kidney resected along with the tumor thrombus. The tumor thrombus was segmented, and the middle segment was slender (red arrow). (**B**) The kidney dissected along the opposite side of the hilum. A round lesion (RCC) was observed on cross-section; the transverse section area was grayish brown and the focal area closely adhered to the perirenal fat. (**C**,**D**) The kidney dissected along the hilar side. A fat-rich lesion (AML) was found (blue arrow); it had a cord-like growth, and its cross-section area appeared golden brown.

The excised tissue was dissected by both the surgeon and pathologist simultaneously, and a golden, slender tumor thrombus was attached to the renal hilum in the gross specimen (Figure 3A). A solid nodule (the bigger one) measuring 2.3 cm × 1.8 cm × 1.8 cm and protruding to the renal capsule was observed (Figure 3B). Another solid lesion (the smaller one) measuring 1.1 cm × 2.5 cm × 0.6 cm was observed adjacent to the renal pelvis and renal sinus (Figure 3C). The tumor thrombus was derived from the smaller lesion and was not related to the bigger one.

Microscopic examination revealed that the bigger lesion was a clear cell renal cell carcinoma with WHO/ISUP nuclear grading of 2–3, and the smaller one was an AML, which was mainly composed of fat. Immunohistochemical staining results revealed the following: Melan-A (positive), HMB45 (positive), and Cathepsin K (suspected positive). 

### 2.6. Follow-Up Situation

The postoperative course was uneventful, and the patient was discharged from the hospital on the fifth day. The patient was asked to return to the clinic for follow-up at three months, six months, and one year postoperatively for a medical history, physical examination, laboratory tests (routine blood, routine urine, and renal function), and imaging tests including ultrasound (3 months, 6 months, and 1 year) and CT (6 months and 1 year). During the one-year follow-up period, no signs of recurrence or metastatic disease were observed. No hematuria, fever, renal function decline, tumor recurrence, or other adverse events were observed. 

## 3. Discussion

According to the WHO Oncology classification, renal AML is divided into two types based on morphology, growth pattern, immunohistochemistry, and genetics [15]. Classic AML is defined as a benign mesenchymal tumor consisting of mature adipose tissue, spindle cells, epithelioid smooth muscle cells, and thick-walled blood vessels. Epithelioid angiomyolipoma (EAML) is defined as a mesenchymal tumor with malignant potential, characterized by epithelioid cell hyperplasia and invasive, destructive growth based on the structure of classic AML. EAML can be aggressive, with local invasion, distant metastasis, and higher recurrence and mortality rates [16]. In the present case, fat-rich AML was observed on CT, and no obvious characteristics of EAML such as proliferation of epithelioid cells with atypia, mitotic activity, necrosis, hemorrhage, and vascular invasion [17] were found on histopathologic examination. Therefore, a diagnosis of EAML was excluded.

The AML location in this patient was the renal hilum, where fat is abundant, and the lesion itself was rich in fat as in classic AML; thus, it was challenging to distinguish it from the surrounding tissue on CT. In addition, the AML lesion was relatively small, and the imaging signal was consistent with the tumor thrombus, together with the interference of surrounding tissues, which we considered the initial part of the tumor thrombus. Preoperatively, we found that the tumor thrombus did not show spatial continuity with the RCC, and there was a “non-thrombotic zone” on the path—we termed it a “ghost thrombus”. Postoperative dissection of the gross specimen resolved this confusion.

The early diagnosis of AML mainly depends on imaging technology. Ultrasound is an effective screening method, and CT provides the greatest diagnostic value [9]. AML generally shows as a low-signal intensity on CT. However, if fat and abnormal thick-walled vascular components of AML are not dominant, it is challenging to distinguish it from RCC on CT [16]. In this situation, the fat saturation technique of magnetic resonance imaging (MRI) can improve diagnostic accuracy [18]. In our case, according to CT findings, this case was considered that of a single RCC; thus, further imaging examinations were not conducted for differential diagnoses—this led to a missed diagnosis of AML. If a preoperative T2-weighted MRI was performed in this case, the fat-rich AML lesion might have hyperintense foci within the hypointense background because small fat foci were scattered among muscles and vessels [7]. We performed the contrast-enhanced magnetic resonance angiography (CEMRA) of the IVC preoperatively, thereby providing a panoramic view of IVC involvement. Due to the limitation of the scanning plane, the primary lesion was not scanned, and considering economic reasons, a renal non-contrast enhanced MRI was not performed for the patient, which also promoted the missed diagnosis of AML lesions to a certain extent. In addition, PET/CT is another advanced imaging technique; a previous study found that renal AML demonstrated an increased soft tissue component [19] and very low to low uptake on FDG PET and PET/CT imaging [20], which can be used to identify RCC and AML. However, due to its high price, if only for differential diagnosis rather than the presence of metastases, its clinical benefit is questionable.

Previous consecutive imaging follow-up studies have found that some AMLs have no or negligible growth, which is a benign behavior, such as a case reported by Rumancik [21]. Therefore, if a small and asymptomatic mass can be clearly identified as AML, it can only be managed with regular monitoring [22]. Symptomatic AML or tumors measuring > 4–6 cm are widely accepted as indications for therapeutic treatments, including surgical resection, selective transarterial embolization, and mammalian target of rapamycin (mTOR) inhibitors [23]. Although most patients treated surgically undergo nephron-sparing surgery, radical nephrectomy is recommended if a tumor thrombus of the IVC is present, regardless of the presence of symptoms; Salami reported a particularly successful case of radical resection [16]. As reported in this case, since the tumor thrombus has extended to the atrium, radical resection should be performed to prevent cardiopulmonary embolization events. Given the location of AML, it is difficult to perform partial nephrectomy and perfectly manage the vasculature without complications, not to mention the concurrence of tumor thrombus.

Cardiopulmonary bypass with deep hypothermic circulatory arrest is recommended for most patients with a supraphrenic or right atrial tumor thrombus [24]. Hideaki et al. suggested that in the case of a tumor thrombus extending to the right atrium, a comprehensive approach of transesophageal echocardiography monitoring of the cardiac thrombus and cooperation with vascular and cardiac surgeons should be considered [25]. In the present case, preoperative imaging revealed that the tumor thrombus had a linear morphology. In addition, previous studies and prediction models supported our conclusion that the tumor thrombus in this patient had no adhesion or invasion of the IVC wall [26]. Therefore, cardiopulmonary bypass was not performed. A preoperative multidisciplinary consultation was agreed upon, and vascular and cardiac surgeons were available to assist when necessary. However, it is necessary to be aware of the possibility of pulmonary embolism caused by a dislodged tumor thrombus, especially on the premise that preoperative ultrasound showed a slender middle segment of the tumor thrombus. Transesophageal ultrasound played a monitoring role intraoperatively, and it was confirmed that there was no residual tumor thrombus (in situ) or detachment (heart) after completion of thrombectomy. Compared to the approved retroperitoneal approach, the transperitoneal approach has the following advantages: large space to facilitate operation, control of renal artery of flexible choice, and intraoperatively open to facilitate the transit, including emergency situations such as bleeding. So, the final decision was the transperitoneal approach with transesophageal echocardiographic monitoring for cardiac embolus without cardiopulmonary bypass.

Que et al. reported 45 cases of AML with a tumor thrombus and discussed diagnosis and treatment strategies [11]. They concluded that the tumor thrombus of AML is mainly composed of adipose tissue, which is soft, irregular, and easy to remove. It is essential to evaluate tumor thrombus length, blood supply, superior and inferior planes, and venous invasion to improve preoperative safety and reduce the occurrence of cardiopulmonary thromboembolism. Preoperative imaging revealed that the tumor thrombus was a discontinuous strip and beaded, and the CT value was negative. Transesophageal ultrasonography showed a beaded echo in the IVC without apparent adhesion to the vessel wall, extending to the entrance of the right atrium. Patients who undergo IVC reconstruction are at high risk for postoperative thrombotic events, and therefore, low-molecular-weight heparin anticoagulation was routinely used in this patient during the perioperative period. Furthermore, abdominal cavity effusion is also prone to complications; thus, we placed an abdominal cavity drainage tube and conducted daily monitoring quantity of flow. In order to prevent potential blood loss after surgery, we regularly reviewed routine blood after surgery and prepared allogeneic blood in advance.

The kidney removed during surgery had two lesions: AML in the renal hilum and RCC near the renal capsule, and the tumor thrombus originated from AML. It was only then that the true nature of the “ghost tumor thrombus” was revealed, and the spatial heterogeneity between the initial part of the tumor thrombus and RCC on preoperative imaging was reasonably explained. The association between RCC and AML in the same kidney has been reported in patients with TS and has been revealed on histopathological examination [27]. Our patient had no evident clinical manifestations of TS, including seizures, mental retardation, or facial angiofibromas [28]. Additional foci of AML in the unilateral nephrectomy specimen were not found; thus, AML in this patient was considered sporadic.

The most reliable method for differential diagnosis of AML and RCC is the use of immunohistochemical markers [9]. RCC is immunoreactive to PAX-8, cytokeratin, and EMA, and these are negative in AML [8]. AML generally shows co-expression of melanocytic markers such as HMB-45 and Melan-A, and also has positive expression for myoid markers such as SMA, MSA, calponin, and/or desmin [8]. In this patient, immunohistochemistry of the hilar lesion revealed positivity for both melanocyte markers, which supported the diagnosis of AML. Further, Cathepsin K was suspected to be present. A previous study suggested that Cathepsin K might degrade extracellular membrane proteins and disrupt the elastic layer of blood vessels to promote cancer invasion and progression [29]. Its expression and activity have been shown to be increased in patients with mesenchymal or epithelial tumors [29]. Therefore, Cathepsin K may be partly responsible for thrombus formation in this patient; however, this hypothesis requires further investigation.

A limitation in the management of this patient was the lack of genetic testing. According to previous reports [9], mutations in the TSC1/TSC2 gene and translocation of the TFE3 gene lead to hyperactivation of the mTOR complex in AML patients. Targeted therapy with mTOR inhibitors is a strategy to prevent further tumor progression and promote regression of existing tumors [30]. The mTOR pathway has also been shown to play a role in the development and progression of RCC, and the United States Food and Drug Administration has approved mTOR inhibitors as therapeutic agents [31]. However, studies have also found that their benefits are relatively limited, and some responders often develop drug resistance [32]. The lack of genetic testing made it uncertain whether the patient would benefit from targeted therapy. The clinical staging of RCC was T1a, which is an early stage, with no metastases observed in various preoperative examinations. Therefore, adjuvant therapy, including targeted therapy, was not administered.

## 4. Conclusions

We present a patient who had renal AML with a tumor thrombus in the IVC and right atrium, accompanied by RCC. If clinicians encounter unconventional clinical manifestations or imaging characteristics, it is necessary to be alert to the possibility of rare diseases, especially the coexistence of multiple rare conditions. Multidisciplinary treatment is advantageous for the management of rare diseases, and early imaging examination and treatment planning are necessary for favorable outcomes. For a level 4 tumor thrombus with no infringement of the venous wall, adoption of robot-assisted minimally invasive surgery, without extracorporeal circulation technology, is feasible. This may reduce intraoperative bleeding and postoperative hospitalization days.

## Data Availability

The data presented in this study are available on request from the corresponding author (because the data are not publicly available due to privacy or ethical restrictions).

## Supplementary Materials

The following supporting information can be downloaded at: https://www.mdpi.com/article/10.3390/medicina60081293/s1, Video S1: Dynamic images of abdominal contrast-enhanced computed tomography, including coronal, sagittal, and horizontal views; Video S2: The key steps of resection of the tumor thrombus in the inferior vena cava during operation; CARE: The CARE-checklist with line number was filled out.
