# Supplementary material for: Renal Angiomyolipoma with Tumor Thrombus in the Inferior Vena Cava and Right Atrium Accompanied by Renal Cell Carcinoma: A Case Report
Source: Medicina (Kaunas). 2024 Aug 10;60(8):1293. doi: 10.3390/medicina60081293 (PMC11356200; doi:10.3390/medicina60081293)
Supplement: Supplementary file 1 [file medicina-60-01293-s001.zip › CARE.pdf]

CARE Checklist of information to include when writing a case report

CARE case report guidelines

(cc) BY-NC-ND

Topic	Item	Checklist item description	Reported on Line
**Title**	1	The diagnosis or intervention of primary focus followed by the words "case report"	2
**Key Words**	2	2 to 5 key words that identify diagnoses or interventions in this case report, including "case report"	29
**Abstract (no references)**	3a	Introduction: What is unique about this case and what does it add to the scientific literature?	12
	3b	Main symptoms and/or important clinical findings	15
	3c	The main diagnoses, therapeutic interventions, and outcomes	19
	3d	Conclusion—What is the main "take-away" lesson(s) from this case?	22
**Introduction**	4	One or two paragraphs summarizing why this case is unique (**may include references**)	51
**Patient Information**	5a	De-identified patient specific information	64
	5b	Primary concerns and symptoms of the patient	65
	5c	Medical, family, and psycho-social history including relevant genetic information	NA
	5d	Relevant past interventions with outcomes	NA
**Clinical Findings**	6	Describe significant physical examination (PE) and important clinical findings	69
**Timeline**	7	Historical and current information from this episode of care organized as a timeline	NA
**Diagnostic Assessment**	8a	Diagnostic testing (such as PE, laboratory testing, imaging, surveys).	71
	8b	Diagnostic challenges (such as access to testing, financial, or cultural)	NA
	8c	Diagnosis (including other diagnoses considered)	127
	8d	Prognosis (such as staging in oncology) where applicable	NA
**Therapeutic Intervention**	9a	Types of therapeutic intervention (such as pharmacologic, surgical, preventive, self-care)	103
	9b	Administration of therapeutic intervention (such as dosage, strength, duration)	NA
	9c	Changes in therapeutic intervention (with rationale)	NA
**Follow-up and Outcomes**	10a	Clinician and patient-assessed outcomes (if available)	132
	10b	Important follow-up diagnostic and other test results	137
	10c	Intervention adherence and tolerability (How was this assessed?)	NA
	10d	Adverse and unanticipated events	NA
**Discussion**	11a	A scientific discussion of the strengths AND limitations associated with this case report	256
	11b	Discussion of the relevant medical literature **with references**	143
	11c	The scientific rationale for any conclusions (including assessment of possible causes)	275
	11d	The primary "take-away" lessons of this case report (without references) in a one paragraph conclusion	275
**Patient Perspective**	12	The patient should share their perspective in one to two paragraphs on the treatment(s) they received	NA
**Informed Consent**	13	Did the patient give informed consent? Please provide if requested	Yes ☑ No ☐
